# Treatment With a Flavonoid-Rich Fraction of Bergamot Juice Improved Lipopolysaccharide-Induced Periodontitis in Rats

**DOI:** 10.3389/fphar.2018.01563

**Published:** 2019-01-17

**Authors:** Enrico Gugliandolo, Roberta Fusco, Ramona D’Amico, Matteo Peditto, Giacomo Oteri, Rosanna Di Paola, Salvatore Cuzzocrea, Michele Navarra

**Affiliations:** ^1^Department of Chemical, Biological, Pharmaceutical and Environmental Sciences, University of Messina, Messina, Italy; ^2^Department of Biomedical Sciences, Dentistry and Morphological and Functional Images, University of Messina, Messina, Italy; ^3^Department of Pharmacological and Physiological Science, Saint Louis University School of Medicine, St. Louis, MO, United States

**Keywords:** periodontitis, Bergamot juice, *Citrus bergamia*, inflammation, lipopolysaccharides, periodontal diseases

## Abstract

**Objective:** In this study, we investigated the effects of a flavonoid-rich fraction of Bergamot juice (BJe) in rats subjected to experimental periodontitis induced by a single intragingival injection of lipopolysaccharides (LPS).

**Main Methods:** Periodontitis was induced by a single intragingival injection of 1 μl LPS (10 μg/μl) derived from *Salmonella typhimurium* in sterile saline solution. The injection was made in the mesolateral side at the interdental papilla between the first and the second molar. Fourteen days after LPS injection, we performed radiographic analyses and then we surgically removed the gingivomucosal tissue surrounding the mandibular first molar for histological, immunohistochemical and molecular analysis.

**Results:** LPS significantly induced oedema, tissue damage and increased neutrophil infiltration. At molecular level, we found increased NF-κB translocation as well as raised both TNF-α and IL-1β expression, other than modulation of apoptosis-associated proteins. Moreover, the increased myeloperoxidase activity was associated with up-regulation of adhesion molecules. Immunohistochemical analysis for nitrotyrosine and poly ADP-ribose displayed an intense staining in the gingivomucosal tissue. Oral administration of BJe for 14 consecutive days reduced tissue injury and several markers of gingival inflammation including nuclear NF-κB translocation, cytokines expression, myeloperoxidase activity and the expression of some adhesion molecules such as ICAM and *P*-selectin. BJe also decreased both nitrosative stress and PARP positive staining. Moreover, it caused down-regulation of Bax and up-regulation of Bcl-2 expression.

**Conclusion:** Our findings demonstrate that BJe improves LPS-induced periodontitis in rats by reducing the typical markers of inflammation, thus suggesting its potential in the treatment of periodontal diseases.

## Introduction

Periodontal disease is a major health problem that affects a great portion of the world population (up to 90%) (Thomas and Puleo, 2011). Periodontitis is an inflammatory disease involving the supporting structures of the teeth, and is caused by an accumulation of periodontopathic bacteria at the gingival sulcus. This provokes inflammation of periodontium, that in turn causes damage of soft connective tissue surrounding both teeth and bone, thus leading to the loss of teeth supporting structures. Moreover, periodontal disease has been associated with cardiovascular and pulmonary diseases, stroke, diabetes, and adverse pregnancy outcomes, although the causal relations are still unknown (Kinane et al., 2017; Nazir, 2017). Depth of periodontal pockets, amount of marginal bone loss, number of teeth with furcation development and degree of attachment loss are the main characteristics of periodontitis (Pihlstrom et al., 2005). The etiopathogenesis of periodontal disease is influenced by host response, presence of pathogenic bacteria and risk factors (Kinane et al., 2017). The latter include external (such as smoking) and systemic influences (such as diabetes mellitus), intrinsic and local factors like gender, race, oral hygiene, age, socioeconomic status, systemic health status and use of medications. Bacteria responsible for periodontitis contain lipopolysaccharides (LPS) which cause periodontal tissues inflammation through the activation of multiple mechanisms: neutrophils, T lymphocytes, leucocytes, plasma cells and the overproduction of reactive oxygen species (ROS) and pro-inflammatory mediators, including prostaglandins and cytokines (Barksby et al., 2009). The inflammatory process involves the stimulation of fibroblasts by interleukin-1β (IL-1β), the increase of collagen breakdown and the rise of osteoclast activity by tumor necrosis factor-α (TNF-α), resulting all together in bone resumption (Kinney et al., 2007).

In the last decades, we attended a comeback to natural remedies to treat several illnesses (Micali et al., 2014; Miroddi et al., 2014; Cirmi et al., 2016c), and growing attention has been dedicated to the role of antioxidants in periodontitis (Muniz et al., 2015; Ramesh et al., 2016). In this line, a number of studies indicated that natural remedies could be used in the prevention and treatment of periodontal diseases with lesser adverse effects than the synthetic drugs (Kumar et al., 2013; Moro et al., 2018).

*Citrus aurantium ssp. Bergamia* Risso & Poiteau also known as *Citrus bergamia* or Bergamot is a small tree appertaining to the family of Rutaceae, whose fruit is primarily exploited for the extraction of its essential oil, employed in perfume industry and aromatherapy (Navarra et al., 2015b; Mannucci et al., 2017) as well as studied for its beneficial effects (Navarra et al., 2015a; Cirmi et al., 2016a; Mannucci et al., 2017). Bergamot Juice (BJ) comes from squeezing the fruits endocarp, and for long time has been considered as a waste product. Recently, several studies have investigated its antimicrobial effects (Filocamo et al., 2015), as well as its anti-cancer activity, both *in vitro* (Delle Monache et al., 2013; Visalli et al., 2014; Ferlazzo et al., 2016b) and *in vivo* (Navarra et al., 2014) models. Moreover, the flavonoid-rich fraction of BJ (BJe) is clinically used as hypolipidemic remedy (Toth et al., 2015; Mannucci et al., 2017). In recent times, some studies have showed that bergamot derivatives possess anti-inflammatory properties (Ferlazzo et al., 2016a). In this field, BJe has been reported to possess antioxidant (Ferlazzo et al., 2015, 2016c) and anti-inflammatory activities both in cell cultures (Risitano et al., 2014; Curro et al., 2016) and in animal models (Impellizzeri et al., 2015, 2016).

On these bases, the present study was designed to investigate the anti-inflammatory effect of BJe on animal model of periodontitis induced by LPS in rats, through both histopathological and molecular analysis.

## Materials and Methods

### Materials

Unless otherwise indicated, all materials were acquired from Sigma-Aldrich Company Ltd. (Poole, Dorset, United Kingdom). All stock solutions were set in non-pyrogenic saline solution (0.9% NaCl; Baxter Healthcare Ltd., United Kingdom).

### Drug

**FIGURE 1:**
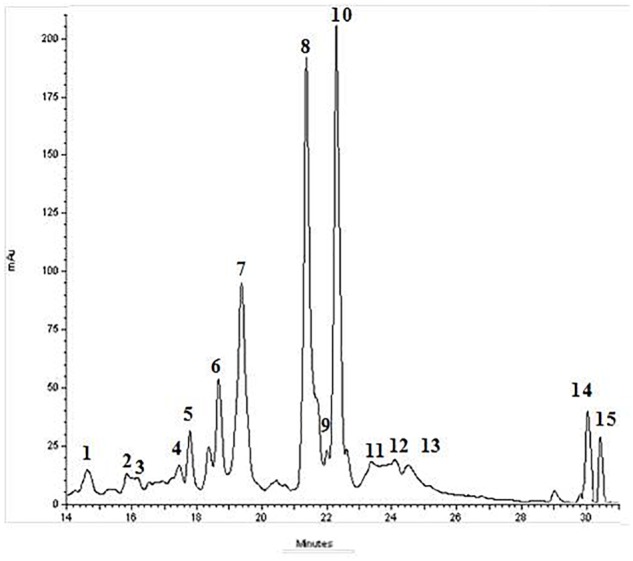
Typical HPLC-DAD chromatograms of BJe recorded at 278 nm. UV-Vis spectrum of the eluted compounds was monitored between 200 and 800 nm. The elution was performed utilizing a linear gradient of acetonitrile in water. Components 1–15 were identified as follows: (1) vicenin-2; (2) stellarin-2; (3) lucenin-2 4’-methyl ether; (4) rhoifolin 4′-glucoside; (5) narirutin 4′-glucoside; (6) eriocitrin; (7) neoeriocitrin; (8) naringin; (9) chrysoeriol 7-*O*-neohesperidoside; (10) neohesperidin; (11) neodiosmin; (12) melitidin; (13) brutieridin; (14) naringenin; (15) hesperetin.

BJe was from the “Agrumaria Corleone” company (Palermo, Italy) which processed bergamot fruits harvested in orchards of Reggio Calabria Province (Italy). From its liquid form, BJe was transformed into dry powder by lyophilization, and then stored at -20°C. Finally, aliquots of BJe were defrosted and their content dissolved into non-pyrogenic saline solution just prior to be given to the animals, as already reported (Impellizzeri et al., 2015). In this experimental research, we employed the same BJe already used in other studies (Risitano et al., 2014; Visalli et al., 2014; Ferlazzo et al., 2015, 2016c; Impellizzeri et al., 2015, 2016; Curro et al., 2016). However, before starting this study, we have repeated the quali-quantitative analysis of flavonoids present in BJe by high performance liquid chromatography system (HPLC; Figure 1), following the procedure described by Gattuso and coworkers (Gattuso et al., 2007). The present results reflect the chemical composition of BJe previously reported (Risitano et al., 2014; Visalli et al., 2014; Ferlazzo et al., 2015). The main flavonoids of BJe (in order of concentration expressed in mg/g) were neohesperidin (96.24), naringin (93.73), melitidin (65.82), hesperetin (52.02), neoeriocitrin (51.80), and naringenin (39.7).

### Animals

Sprague-Dawley male rats (200–230 g, Harlan, Italy) were housed in an animal facility with food and water *ad libitum*, minimizing stress conditions. The University of Messina Review Board approved the study for the care of animals. Experiments were performed according to both the new legislation for the protection of animals used for scientific purposes (Directive 2010/63/EU) and ARRIVE guidelines.

The minimum number of animals for each group (*n* = 10) was calculated with the *a priori* power analyses statistical test of the G-power software, as this statistical test provides an efficient method to determine the sample size needed to carry out the experiment before the experiment itself is actually conducted. Rats were randomized with the technique of “simple randomization” (Ivers et al., 2012).

### LPS-Induced Periodontitis

Method of Gugliandolo et al. (2018) was used to induce periodontitis. Animals were lightly anesthetized with sodium pentobarbitone (35 mg/kg), and then periodontitis was induced by a single intragingival injection of 1 μl LPS (10 μg/μl) derived from Salmonella typhimurium (Sigma-Aldrich) in sterile saline solution. The injection was made in the mesolateral side at the interdental papilla between the first and the second molar. It was performed slowly and the needle kept in place for some seconds after the injection to guarantee that LPS was not lost through needle extraction. In order to control food intake/masticatory behavior, animals were weighed at regular time intervals, while to evaluate periodontitis lesions, rats were sacrificed at 14 days after LPS injection. We performed LPS injection bilaterally so we have 20 samples/group and used five for each analysis.

### Experimental Groups

Rats were randomly divided in the following groups (*n* = 10 for each):

– Group 1: LPS + saline group: rats were subjected to LPS-induced periodontitis as described above (*N* = 10).
– Group 2: LPS + BJe group: same as the LPS + saline group, and BJe (20 mg/kg) was administered by oral gavage every 24 h for 14 days, starting from 1 h after the injection of LPS (*N* = 10).
– Group 3: Sham + saline group: animals received a single intragingival injection of saline solution with an identical surgical procedures described for LPS group (*N* = 10).
– Group 4: Sham + BJe group: same as the Sham + saline group, and BJe (20 mg/kg) was administered by oral gavage every 24 h (1 h after LPS-injection) for 14 days (*N* = 10).

The tested dose for BJe was chosen in agreement with previous findings (Impellizzeri et al., 2015, 2016).

### Radiography

Radiographic analyses of mandibles of rats belonging to the four experimental groups above were performed by an X-ray machine (Bruker MS FX Pro, Billerica, MA, United States). The X-ray tube was operated at 30 kW, with a current of 6 mA, for 0.01 s, and the source-to-sensor distance was 50 cm. Fourteen days after the beginning of experiments (injection of LPS with or without BJe treatment), we evaluated the dental alveolar bone level defined as the distance from the cemento-enamel junction (CEJ) to the maximum coronal level of the alveolar bone crest (CEJ–bone distance) using IMAGE J processing software (Image J software, National Institutes of Health, Bethesda, MD, United States) (Gugliandolo et al., 2018).

### Measurement of Vascular Permeability

For vascular permeability, rats received the Evans blue (2.5% dissolved in saline, at a dose of 50 mg/kg) through a femoral venous catheter. To extract the extravasated Evans blue, gingivomucosal tissues were embedded for 48 h at room temperature with 1 mL formamide. Spectrophotometric determination was assessed at 620 nm and evaluated as μg/g gingivomucosal tissue.

### Histopathological Examination

Gingivomucosal tissues were surgically removed 14 days after starting the experiments (injection of LPS followed or not by BJe treatment). Samples were fixed in 10% (w/v) PBS-buffered formaldehyde solution at 25°C for 24 h, then dehydrated by graded ethanol solutions and subsequently embedded in Paraplast (Sherwood Medical, Mahwah, NJ, United States). From each paraffin block 7 μm sections were prepared deparaffinised with xylene and colored with hematoxylin and eosin. Five serial sections for each animal were investigated using Axiovision Zeiss (Milan, Italy) microscope. All sections were investigated using Axiovision Zeiss (Milan, Italy) microscope. All the histological studies were executed in a blinded fashion. A histological injury score for gingivomucosal tissue was determined using a semiquantitative scale that measures the following morphological criteria: 0, normal gingivomucosal tissue; grade 1, minimal oedema or infiltration; grade 2, moderate oedema and inflammatory cell infiltration without obvious damage to gingivomucosal architecture; grade 3, severe inflammatory cell infiltration with obvious damage to gingivomucosal architecture. In order to evaluate fibrosis degree, gingivomucosal sections were positioned longitudinally from the teeth crowns and stained with the Masson trichrome stain, according to the manufacturer’s instructions (Bio-Optica, Milan, Italy).

### Measurement of Cytokines

In order to evaluate tissue levels of TNF-α and IL-1β, gingivomucosal tissues were homogenized in 2 mmol/L of PBS containing phenylmethylsulfonyl fluoride (Sigma-Aldrich). The assay was performed using a colorimetric kit (Calbiochem-Novabiochem Corporation, United States), according to manufacturer’s protocol. All cytokine determinations were carried out in duplicate serial dilutions. Results are showed as pg/mL.

### Myeloperoxidase Activity

Myeloperoxidase activity, an indicator of polymorphonuclear leukocyte (PMN) accumulation, was determined in gingivomucosal tissue, as previously described (Mullane et al., 1985). Myeloperoxidase activity was defined as the quantity of enzyme degrading 1 μmol/min of peroxide at 37°C and was expressed in milliunits/g of wet tissue.

### Immunohistochemical Localization of Nitrotyrosine, PAR, ICAM and *P*-Selectin

After 14 days of LPS injection followed or not by BJe treatment, gingivomucosal tissues were fixed in 10% (w/v) PBS-buffered formaldehyde and embedded in paraffin. From each paraffin block 7 μm sections were prepared in which, after deparaffinization, endogenous peroxidase was quenched with 0.3% (v/v) hydrogen peroxide in 60% (v/v) methanol for 30 min. The slides were permeabilized with 0.1% (w/v) Triton X-100 in PBS for 20 min. Non-specific adsorption was decreased by incubating sections in 2% (v/v) normal goat serum in PBS for 20 min. Endogenous avidin or biotin binding sites were blocked by sequential incubation for 15 min with avidin and biotin (Vector Laboratories, Burlingame, CA, United States), respectively. Sections were incubated overnight with the following antibodies dissolved in PBS (v/v): anti-nitrotyrosine (Merck Millipore, Milan, Italy; 06-284), or anti-PARP (Santa Cruz Biotechnology, H-250: sc-7150, 1:350), or anti-ICAM (Santa Cruz Biotechnology Inc., Dallas, TX, United States; C-20: sc-8439, 1:460), or anti-*P*-selectin (Santa Cruz Biotechnology, G-5: sc-6941, 1:460). Sections were then washed with PBS, and incubated with secondary antibody. Specific labeling was detected with a biotin-conjugated goat anti-rabbit IgG and avidin-biotin peroxidase complex (Vector Laboratories). To verify antibody-binding specificity, control slices were incubated with only primary antibody or secondary antibody, neither of which gave positive staining. Images were collected using a Zeiss microscope and Axio Vision software. For graphic display of densitometric analyses, the % of positive staining (brown staining) was measured by computer-assisted color image analysis (Leica QWin V3, United Kingdom). The percentage area of immunoreactivity (determined by the number of positive pixels) was expressed as % of total tissue area (red staining) within five random fields at 40x magnification. In particular, firstly the colors of the images that have been stained to the molecule of interest were defined. Once these colors were defined, they were automatically detected in all samples. This is a semi-quantitative analysis that measures areas and not intensities (Hernandez-Aguilera et al., 2015).

### Western Blot Analysis for IκBα, NF-κB, Bax and Bcl-2

Gingivomucosal tissues from each rat employed in this study were suspended in extraction buffer A containing 0.15 μM pepstatin A, 0.2 mM PMSF, 1 mM sodium orthovanadate and 20 μM leupeptin, homogenized at the highest setting for 2 min, and then centrifuged at 1000 × *g* at 4°C for 10 min. Cytosolic fraction was represented by the supernatants. The pellets, containing enriched nuclei, were re-suspended in buffer B containing 150 mM NaCl, 1% Triton X-100, 1 mM EGTA, 10 mM Tris–HCl pH 7.4, 0.2 mM PMSF, 1 mM EDTA, 0.2 mM sodium orthovanadate and 20 μm leupeptin. After centrifugation at 15.000 *g* and 4°C for 30 min, the nuclear protein contained in the supernatants were stored at -80°C for further analysis. The levels of IκB were measured in the cytosolic fraction, while NF-kB p65 levels were quantified in nuclear one. Membranes were blocked with 1× PBS, 5% (w/v) non-fat dried milk at room temperature for 40 min, and subsequently probed with the following antibodies: specific anti-IκBα (Santa Cruz Biotechnology, C-21: sc-371, 1:550), or anti-Bax (Santa Cruz Biotechnology, P-19: sc-526, 1:530), or anti-Bcl-2 (Santa Cruz Biotechnology, N-19: sc-492, 1:510), or anti-NF-κB p65 (Santa Cruz Biotechnology, F-6: sc-8008, 1:400) dissolved in 1× PBS, 5% w/v dried milk, 0.1% Tween-20 (PMT) at 4°C overnight. Then, membranes were incubated with peroxidase-conjugated bovine anti-mouse IgG secondary antibody or peroxidase-conjugated goat anti-rabbit IgG (1:2000, Jackson ImmunoResearch, West Grove, PA, United States) for 1 h at room temperature. The blots were also probed with primary antibody against β-actin protein or laminin (both 1:10.000; Sigma-Aldrich), employed as internal standards for cytosolic or nuclear fraction, respectively. The relative expression of the protein bands of IκB-α (37 kDa), Bax (23 kDa), Bcl-2 (29 kDa), and NF-κB p65 (65 kDa) was quantified by densitometric scanning of the X-ray films (GS-700 Imaging Densitometer, Bio-Rad Laboratories, Milan, Italy), quantified by densitometric analysis (Molecular Analyst, IBM) and standardized for β-actin or laminin levels.

### Statistical Evaluation

Experimental data reported in both text and figures are expressed as mean ± standard error of the mean (S.E.M.) of the number of animals employed (N). In the experiments including histology or immunohistochemistry, the photos are representative of at least three different experiments. Results were analyzed by one-way analysis of variance (ANOVA) followed by Bonferroni’s *post hoc* test for multiple comparisons. A *P*-value lesser than 0.05 was considered statistically significant.

## Results

### Effects of BJe Administration on Bone Destruction Induced by LPS in Gingival Tissues

**FIGURE 2:**
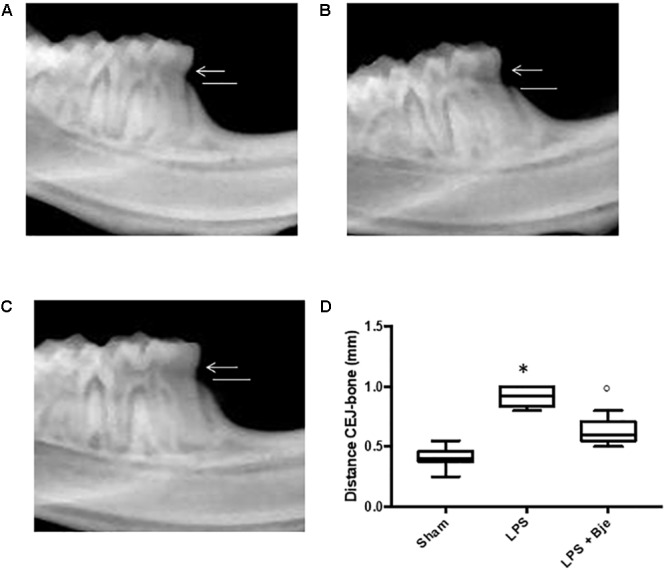
Effect of BJe on alveolar bone loss. After 14 days from the LPS administration, radiographic picture of mandible from LPS-injected rats **(B,D)** showed a bigger distance from the CEJ to the bone, compared to the one of sham group rats **(A,D)**, while mandible from BJe-treated animals showed a shorter distance **(C,D)**. Values reported in the box plot are expressed as mean ± SEM of 10 rats for each group. ^∗^*P <* 0.05 *vs* sham group. °*P <* 0.05 *vs* LPS group.

As shown in Figure 1 the radiographic distance from the CEJ to the bone was significantly larger in the LPS-treated rats (Figures 2B,D) than in the sham-group animals (Figures 2A,D), whereas the CEJ–bone distance in BJe-treated animals was significantly shorter than in LPS-group (Figures 2C,D).

### Histopathological Examination Indicates That BJe Treatment Reduces LPS-Induced Periodontitis

**FIGURE 3:**
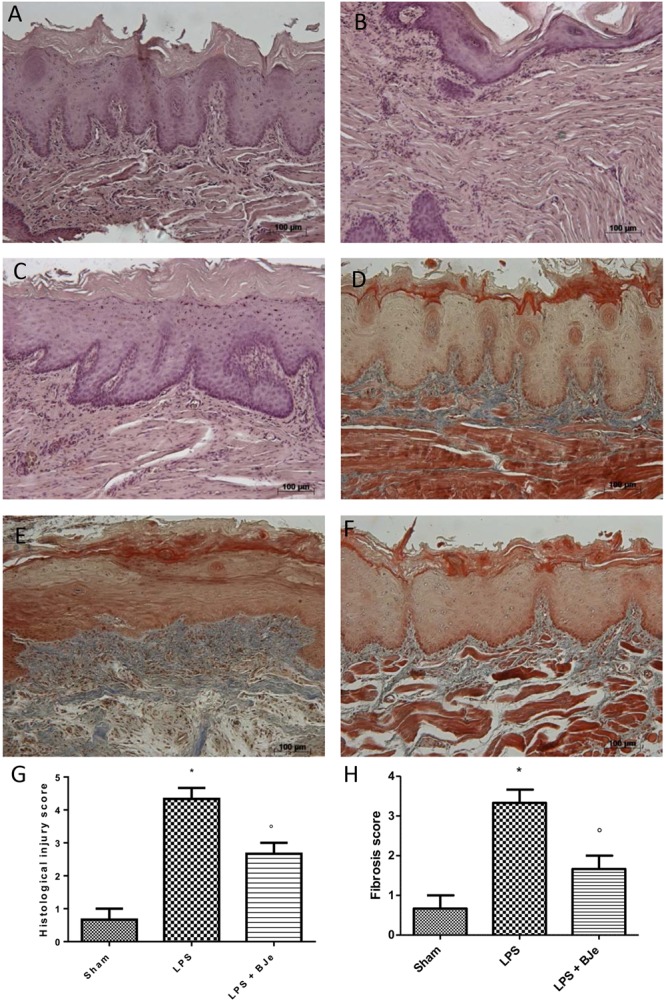
Histopathological aspect of LPS-induced periodontitis in rats. Fourteen days after the start of experiments, gingivomucosal tissues from LPS-injected rats showed oedema, tissue injury and inflammatory cells infiltration **(B,G)** compared to the rats of sham-group **(A,G)**. BJe treatment significantly reduced the inflammatory picture LPS-induced **(C,G)**. Moreover, Masson’s trichrome stain, presented increase in the concentration of collagen fibers in gingivomucosal tissues in vehicle group **(E,H)** when compared with sham group **(D,H)**. Treatment with BJe significantly attenuated collagen formation **(F,H)**. Values are expressed as mean ± SEM (*N* = 10 rats in each group). ^∗^*P <* 0.05 *vs* sham group. °*P <* 0.05 *vs* LPS group.

Figures 3A,B show the histological examination of gingivomucosal tissues collected from animals belonging to the sham and LPS groups, respectively. In the sections from rats injected with LPS (Figure 3B), when compared to those of sham group (Figure 3A) we found a significant increase in oedema and tissue damage (see histological score in Figure 3G) that significantly decreased after BJe administration (Figure 3C, see histological score in Figure 3G). Additionally, Masson’s trichrome stain shown an increase of collagen formation in gingivomucosal tissues sections of LPS injected rats (Figure 3E, see score in Figure 3H) when compared with tissue samples collected from sham rats (Figure 3D, see score in Figure 3H). BJe-treatment reduced the increase of collagen (Figure 3F, see score in Figure 3H).

### BJe Administration Decreases IkB-α and NF-kB Activation Induced by LPS in Gingivomucosal Tissues

**FIGURE 4:**
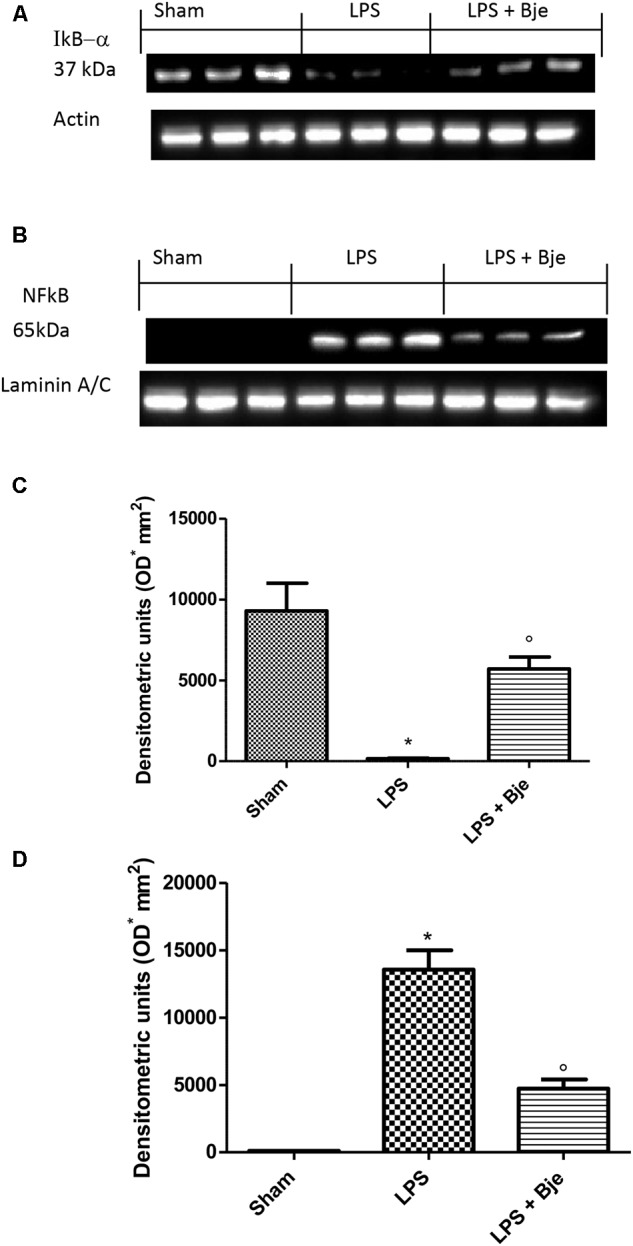
BJe inhibits NF-κB activation. Blot in **(A)** and its densitometric analysis in **(C)** showed that BJe significantly decreased IκB-α degradation **(A,C)**. Blot **(B)** displayed that LPS injection increased NF-κB translocation in the nucleus **(B,D)**, whereas BJe significantly reduced the presence of NF-κB in the nuclear fraction **(B,D)**. Levels of IκB-α and NF-κB presented in the densitometric analyses of protein bands were normalized for β-actin and laminin, respectively. Data reported are presented as mean ± SEM (*N* = 10 rats for each group). ^∗^*P <* 0.05 *vs* sham group. °*P <* 0.05 *vs* LPS group.

In order to assess the molecular mechanism through which BJe reduces the typical signs of experimental periodontitis, first we explored the involvement of NF-κB. It is well-known that IκB-α degradation leads to the nuclear translocation of p65/p50 subunits of NF-κB and its consequential activation. In gingivomucosal tissues from rats of sham-group, we detected basal expression of IκB-αwhich was markedly decreased in samples from animals of LPS-group (Figures 4A,C). Consequentially, LPS increased NF-κB activation in the nuclear fractions of rats belonging to LPS-group as compared to those of the sham-group (Figures 4B,D). Interestingly, BJe treatment inhibited NF-κB activation preventing LPS-induced IκB-α degradation (Figures 4A,C), thus reducing the level of NF-κB in the nucleus (Figures 4B,D) in a statistically significant way. To underline the purity and quality of subcellular fractionation of the proteins, we included actin and laminin as internal standards for cytosolic (Figure 4A) or nuclear (Figure 4B) fraction, respectively.

### BJe Reduces TNF-α and IL-1β Generation, MPO Activity and Plasma Extravasation LPS-Induced

**FIGURE 5:**
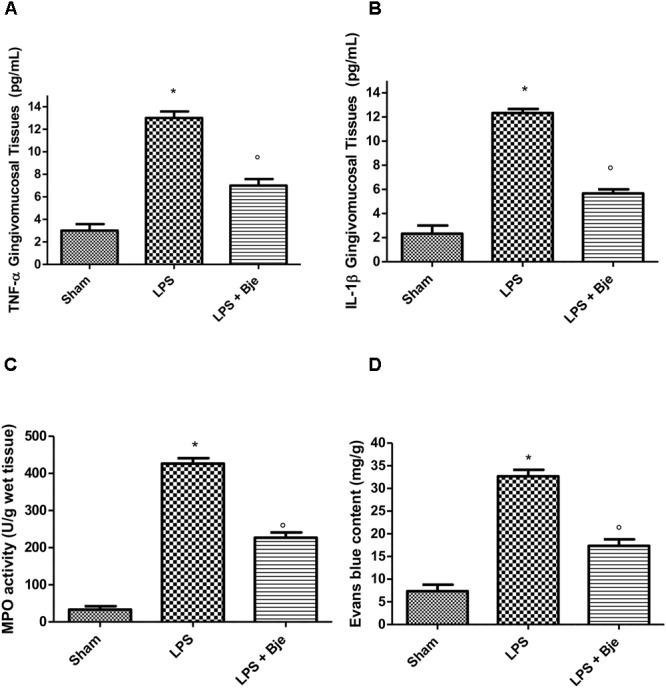
BJe diminishes TNF-α and IL-1β production, MPO activity and plasma extravasation in LPS-induced periodontitis. A significant increase in TNF-α and IL-1β levels was found in gingivomucosal tissues from LPS-injected rats, as compared with the samples of sham-group **(A,B)**, whereas treatment with BJe significantly decreased the generation of these cytokines **(A,B)**. LPS significantly raised myeloperoxidase activity **(C)** compared with those found in sham-group, while BJe significantly reduced these enzymatic activity **(C)**. BJe administration inhibited the increase in Evans blue extravasation induced by LPS **(D)**. Data are mean ± SEM of 10 rats for each group). ^∗^*P <* 0.05 *vs* sham group. °*P <* 0.05 *vs* LPS group.

A significant increase in TNF-α and IL-1β levels was observed in gingivomucosal samples 14 days after LPS injection (Figures 5A,B). In contrast, a significant reduction of these cytokines was found in the tissues of animals injected with LPS and then daily treated with BJe (Figures 5A,B). Moreover, in the LPS-group, myeloperoxidase (MPO) activity was significantly raised in comparison to that detected in the samples from rats of sham-group (Figure 5C). Noticeably, BJe treatment significantly reduced MPO activity (Figure 5C). Furthermore, compared to the sham-group animals, LPS injection significantly increased Evans blue extravasation (Figure 5D), while BJe administration counteracted this growth (Figure 5D).

### Treatment With BJe Counteracts ICAM and *P*-Selectin Expression Induced by LPS-Injection in Gingival Tissue

**FIGURE 6:**
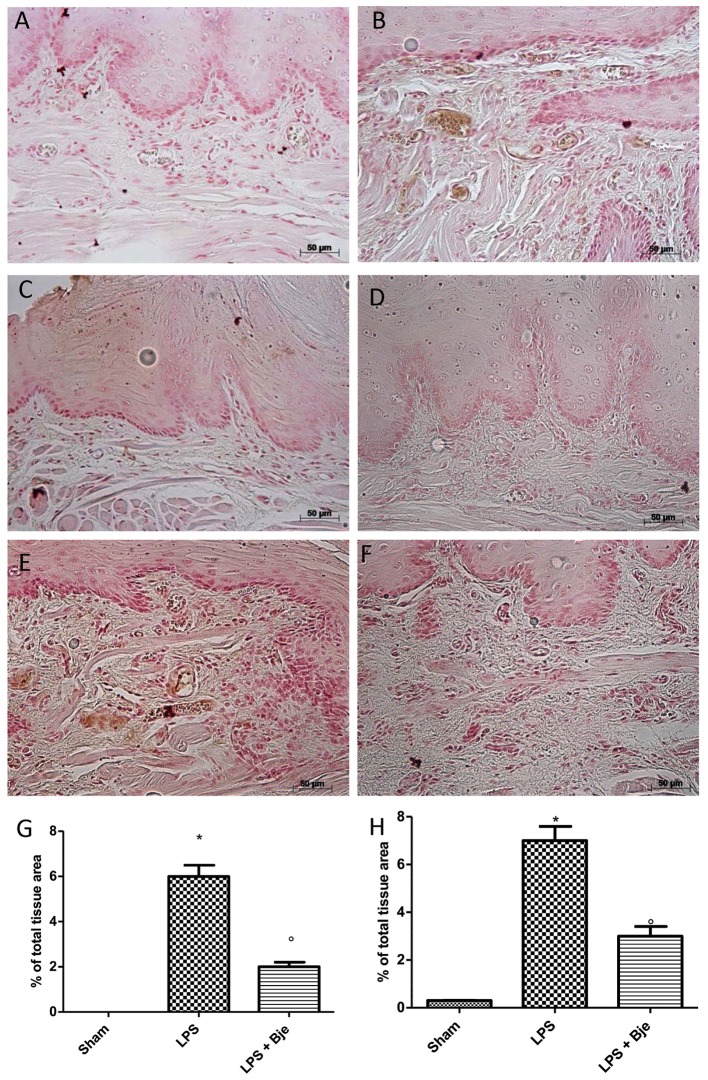
BJe reduces ICAM and *P*-selectin expression. No positive staining for ICAM **(A,G)** or *P*-selectin **(D,H)** was detected in rats of sham group that, instead, was found in tissues from LPS-subjected animals (**B,G** for ICAM; **E,H** for *P*-selectin). In BJe-treated animals we found a decreased staining for ICAM **(C,G)** as well as *P*-selectin **(F,H)**. Values of densitometric analyses are presented as mean ± SEM of 10 rats for each group). ^∗^*P <* 0.05 *vs* sham group. °*P <* 0.05 *vs* LPS group.

Samples of gingivomucosal tissue from rats of sham-group showed a basal staining for ICAM (Figure 6A), while LPS increased this staining (Figure 6B), and BJe significantly down-regulated it (Figures 6C,G). Moreover, immunohistochemical analysis of tissues collected from LPS-treated group showed positive staining for *P*-selectin (Figures 6E,H), as compared to tissues from sham-treated group (Figures 6D,H). Notably, treatment with BJe reduced also this staining (Figures 6F,H).

### BJe Decreases Nitrotyrosine and PARP Expression in LPS-Induced Periodontitis

**FIGURE 7:**
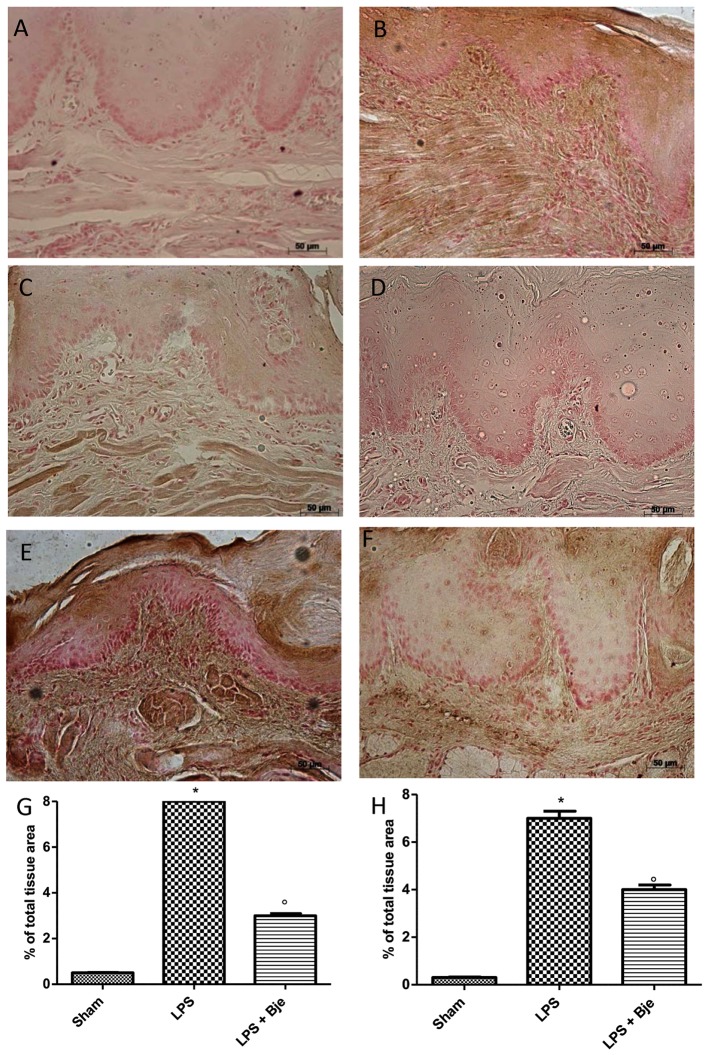
BJe decreases nitrotyrosine and PARP expression. Sections obtained from LPS-injected rats showed intense positive staining for nitrotyrosine **(B,G)** and PARP **(E,H)**, that were found significantly reduced in BJe-treated rats (nitrotyrosine, **C,G**; PARP, **F,H**). No positive immunostaining for nitrotyrosine **(A,G)** and PARP **(D,H)** was observed in gingivomucosal tissues from rat belonging to sham-group. Values in graphs are expressed as mean ± SEM (*N* = 10 rats per each group). ^∗^*P <* 0.05 *vs* sham group. °*P <* 0.05 *vs* LPS.

Immunohistochemical analysis of sections from rats injected with LPS presented positive staining for nitrotyrosine (Figures 7B,G), while reduced positive staining was found in samples of rats treated with BJe for 14 days after the injection of LPS (Figures 7C,G). Moreover, the immunohistological staining for poly ADP-ribosylated proteins revealed a positive staining for the PARP in rats of LPS-group (Figures 7E,H) which was attenuated by the treatments with BJe (Figures 7F,H). No positive staining for nitrotyrosine and poly ADP-robosylated proteins was found in sham group (Figures 7A,D).

### Effects of BJe on Bax and Bcl-2 Expression in Rats Subjected to a Gingival Injection of LPS

**FIGURE 8:**
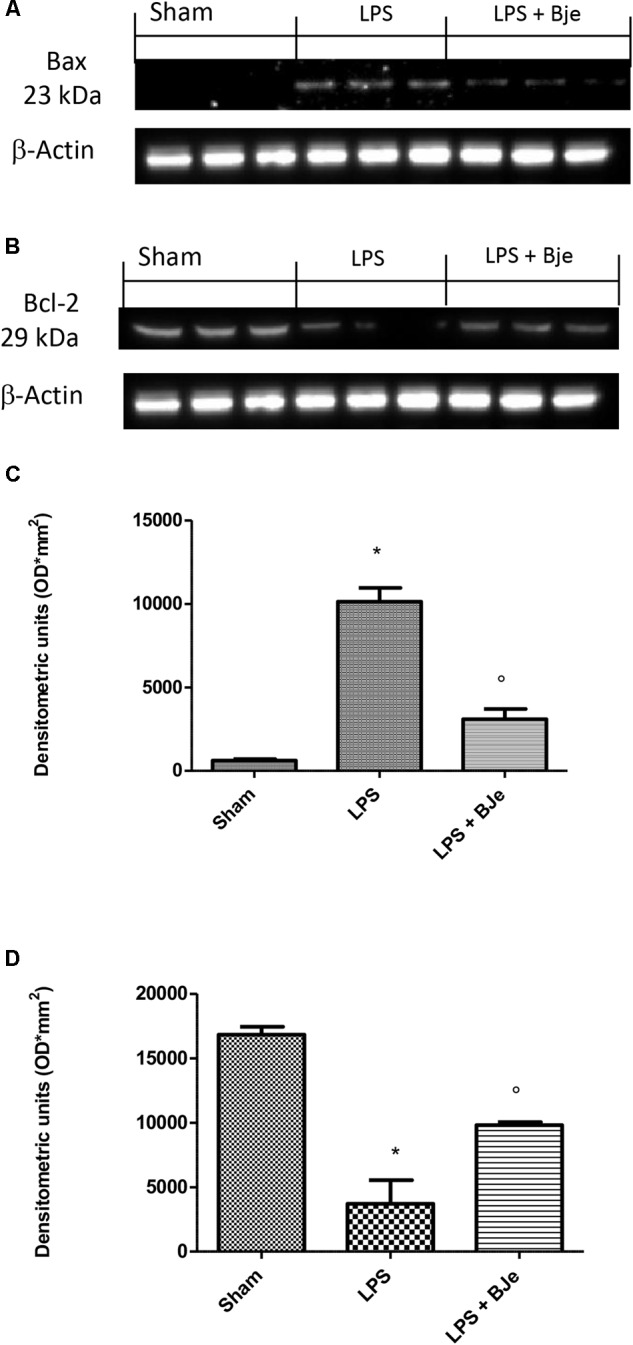
BJe modulates Bax and Bcl-2 expression. Western blot analyses performed on samples of gingivomucosal tissue from LPS-injected rats, compared to sham group, displayed an increase of Bax expression, that was found reduced in BJe-treated rats **(A,C)**. Conversely, LPS decreased expression of Bcl-2 which raised in the rats treated with BJe **(B,D)**. Densitometric analyses of blots were performed normalizing bands for β-actin. Blots in **(A,B)** are representative of three different gels. Data in **(C,D)** are means ± SEM of 10 rats for each group. ^∗^*P <* 0.05 *vs* sham group. °*P <* 0.05 *vs* LPS group.

In order to assess whether damage of gingivomucosal tissues induced by LPS was also associated to apoptosis, we performed Western blot analyses. Figure 8 show that LPS significantly increased expression of Bax (a pro-apoptotic protein; Figures 8A,C) and decreased that of Bcl-2 (anti-apoptotic protein; Figures 8B,D). BJe-treatment significantly down-regulated LPS-induced Bax expression (Figures 8A,C) and significantly increased the levels of Bcl-2 (Figures 8B,D).

## Discussion

Although periodontal pathogens are the main responsible for the etiopathogenesis of periodontitis, there is a growing body of evidences suggesting the pivotal role played by oxidative stress (Kaur et al., 2016; Liu et al., 2017; Wang et al., 2017). *Citrus* flavonoids are acknowledged for their broad spectrum of biological properties, including the well-known antioxidant activity and the capability of interfering with key intracellular pathways that play determinant role in degenerative processes (Cirmi et al., 2016b; Citraro et al., 2016; Fusco et al., 2017). Blignaut and Grobler (1992) suggested that the high fruit consumption (apple, grape, and especially *Citrus* fruit) by the fruit-farm workers improved the periodontal status with respect to the individuals with low or no fruit intake in diet. On the other hand, several studies reported a lower total antioxidant capacity in periodontitis patients compared to healthy subjects (Sculley and Langley-Evans, 2003; Brock et al., 2004). Staudte et al. (2005) suggested that 2 weeks intake of grapefruit leads to increased plasma levels of vitamin C and improves sulcus bleeding scores in patients with chronic periodontitis, especially in smokers. These findings, together with the previous reports on the antioxidant properties of BJe (Marino et al., 2015; Ferlazzo et al., 2016a), led us to investigate its effects in an experimental model of periodontitis in order to evaluate its potentiality and to discover the mechanism of action. To our best knowledge, this is the first study demonstrating the antioxidant and anti-inflammatory activity of a *Citrus* derivative against periodontal disease. In particular, we showed that BJe significantly ameliorated the inflammatory findings of gingivomucosal tissues of rats subjected to LPS-induced periodontitis, downregulating NF-*κ*B activation, pro-inflammatory cytokines levels, ICAM, *P*-selectine, nitrotyrosine and PARP expression as well as apoptosis. In this study, we showed that BJe administration attenuated LPS-induced periodontitis in rats, as suggested by the radiographic analysis of the mesial root surface displaying its ability to reduce periodontal bone-supporting ratio caused by LPS. The protective action of BJe was also evident at tissue level, where we found a histological picture very improved through the extract. As Masson’s trichrome staining shown, LPS injection induced an increase of collagen formation and improved the fibrosis score. It is already reported in literature that simultaneously with collagen destruction, wound repair occurs, which results in fibrosis and scarring coexisting at the foci of inflammation (Bartold and Narayanan, 2006). During periodontal disease despite the degradation of the fibrilar collagen, cells are able to synthesize a new type of collagen, type I trimer which accumulates in the gingiva (Bartold and Narayanan, 2006) or that inflamed human gingiva contains fibroblasts with different phenotype than those from the normal tissue, the myofibroblasts, able to synthesize a large amount of collagen. BJe was able to improve inflammation and fibrosis characterizing periodontal disease. There are evidences showing that both ROS and reactive nitrogen species (RNS) have an important role in the development of the periodontal disease (Trivedi and Lal, 2017). Both ROS and RNS directly modulate NF-κB that in turn regulate gene transcription in many and pathophysiological conditions. Normally, IκBs proteins sequester NF-κB in cytoplasm. In response to several stimuli such as infection, hypoxia, oxidative stress, inflammation and extracellular stimuli, the enzyme IκB kinase phosphorylates the regulatory proteins IκBs. These phosphorylated proteins release the NF-κB dimer, which is free to translocate into the nucleus. In this experimental research we found a significant increase in the translocation of NF-κB in the nucleus of epithelial cells of gingivomucosal tissues from LPS-injected rats, accompanied by IκB-α degradation. Furthermore, it is known that NF-κB has a key role in the activation of many genes answerable for the generation of proteins and mediators in inflammation such as TNF-α and IL-1β. Herein, we showed an increase of TNF-α and IL-1β production 14 days after LPS-injection, confirming the role of these cytokines in the periodontitis pathogenesis. Of note, oral treatment with BJe for 14 consecutive days reduced LPS-induced NF-κB activation and counteracted TNF-α and IL-1β expression in gingivomucosal tissues from LPS-injected rats, suggesting a mechanism through which BJe reduces experimental periodontitis. Overall, these novel findings are in line to those reported about the antioxidant and anti-inflammatory properties of BJe in *in vitro* models. Indeed, previously we showed the antioxidant property of BJe in both abiotic and cell culture models (Ferlazzo et al., 2015), as well as its chelating activity (Ferlazzo et al., 2016c). Moreover, we demonstrated the ability of BJe to inhibit both gene expression and secretion of cytokines such as IL-1β, interleukin-6 (IL-6) and TNF-α induced by LPS in THP-1 monocytes, through a mechanism mediated by the inhibition of NF-κB (Risitano et al., 2014). Others experimental research suggested the involvement of both NF-κB and cytokines in the anti-inflammatory mechanisms exerted by bioactive molecules against periodontitis. For instance, daily curcumin oral gavage inhibited IL-6, TNF-α and PGE_2_ expression in gingival tissues injected with LPS, as well as diminished NF-κB activation and decreased innate immune responses associated with periodontal disease (Guimaraes et al., 2012). Maruyama et al. (2011) suggested that green tea catechins decreased expression of pro-inflammatory cytokines and oxidative stress in experimental periodontal inflammation triggered by topical application of LPS and proteases to the gingival sulcus in rats. Green tea catechins also inhibited nuclear translocation of NF-κB and IL-1β expression induced by LPS in gingival tissue, thus suppressing bone resorption (Nakamura et al., 2010).

It is known that polymorphonuclear neutrophils are engaged into the inflamed tissue where, more than other cells, contributed to generate ROS and RNS, that in turn caused oxidative stress and membrane lipid peroxidation, thus amplifying the inflammatory response. Moreover, it is well known the important role of the adhesion molecules in the polymorphonuclear cells infiltration. In our study, we also showed that treatment with BJe reduced the Evans blue extravasation caused by LPS, as well as reduced MPO activity and infiltration of inflammatory cells in the gingivomucosal tissues of LPS-injected rats. Furthermore, the down-regulation of ICAM and P-selectin expression found in the gingivomucosal tissues from BJe-treated rats suggested a possible mechanism by which it attenuated polymorphonuclear cells infiltration caused by LPS-injection. The role of ROS and RNS in the pathogenesis of periodontal disease, as well as the involvement of PARP in experimental periodontitis have been already described (Di Paola et al., 2004). Herein we showed that BJe administration reduced the LPS-induced nitrosative stress and increased PARP activity in the LPS-injured gingivomucosal tissue. Numerous studies displayed that apoptosis is implicated in the pathogenesis of periodontal disease. Moreover, our results suggested that BJe administration down-regulated expression of the pro-apoptotic protein Bax and up-regulated those of the anti-apoptotic Bcl-2 in gingivomucosal tissue from LPS-injected rats, confirming both the role of apoptosis in the pathogenesis of periodontal disease (Song et al., 2017) and the ability of our extract to modulate the apoptotic machinery. Overall, these findings agree with previous experimental research suggesting the anti-inflammatory activity of BJe in experimental models of both colitis (Impellizzeri et al., 2015) and intestinal ischemia (Impellizzeri et al., 2016), as well as strengthen the potentiality of natural products in periodontal diseases. In this line, very recently, it has been reported that treatment with *Salvia sclarea* extract significantly reduced levels of IL-1β, IL-6 and TNF-α, counteracting lesions of gingival tissue and preserving bone alveolar resorption (Kostic et al., 2017). In recent years, BJe has demonstrated antimicrobial effects as well as antioxidant and anti-inflammatory activity in several *in vitro* and *in vivo* experimental models. In this study, through several methodological approaches, we demonstrate that BJe improves LPS-induced periodontitis in rats, suggesting its potential in the treatment of periodontal disease. Our findings support a novel role of BJe in the treatment of inflammatory pathologies in a contest of a multitarget pharmacological strategy.

## Author Contributions

SC conceived and designed the study. MN performed the chemical analysis of BJe and drafted the manuscript. RF assisted in drafting the manuscript. EG, RF, and RD carried out the experiments. RDP coordinated the study. GO and MP critically revised the manuscript. All authors read and approved the final article.

## Conflict of Interest Statement

The authors declare that the research was conducted in the absence of any commercial or financial relationships that could be construed as a potential conflict of interest. Agrumaria Corleone provided BJe, but had no role in study design, data analysis, or paper’s preparation. The reviewer SA and handling Editor declared their shared affiliation.
